# Dominant-negative activity of the STAT3-Y705F mutant depends on the N-terminal domain

**DOI:** 10.1186/1478-811X-11-83

**Published:** 2013-11-05

**Authors:** Anne Mohr, Dirk Fahrenkamp, Natalie Rinis, Gerhard Müller-Newen

**Affiliations:** 1 Institut für Biochemie und Molekularbiologie, RWTH Aachen University, Pauwelsstraße 30, Aachen 52074, Germany

**Keywords:** JAK-STAT signaling, STAT3, IL-6, STAT3-YF mutant, Dominant-negative, Hyper-IgE syndrome (HIES)

## Abstract

**Background:**

STAT3 is a transcription factor of central importance in chronic inflammation and cancer. In response to cytokine stimulation STAT3 is phosphorylated on a single tyrosine residue at position 705, dimerizes and accumulates in the nucleus to induce target gene expression. The substitution of tyrosine 705 to phenylalanine leads to a dominant-negative STAT3 mutant (STAT3-YF) which influences the activation of WT-STAT3 in stimulated cells through a mechanism that is not completely understood. In this study we analyzed the molecular mechanism of STAT3-YF dominant-negative activity in IL-6-induced STAT3 signaling and the relevance of the N-terminal domain.

**Results:**

Expression of STAT3-YF-YFP impairs tyrosine phosphorylation, nuclear translocation and the transcriptional activity of WT-STAT3 in IL-6-stimulated cells. The fluorescently labelled STAT3-YF mutant binds to a phosphorylated gp130 receptor-peptide comparable to WT-STAT3-YFP. STAT3-YF-YFP forms homodimers as well as heterodimers with WT-STAT3 in the presence and absence of IL-6. The preformed heterodimers in unstimulated cells are detectable by colocalization of STAT3-CFP with STAT3-YF-YFP fused to a nuclear localization signal. STAT3/STAT3-YF heterodimers are not able to bind to DNA in stimulated cells, but the presence of the mutant reduces DNA-binding of WT-STAT3 homodimers. STAT3-YF-ΔN-YFP lacking the N-terminal domain forms no dimers and only marginally affects the activity of WT-STAT3.

**Conclusion:**

Our findings demonstrate that dominant-negative STAT3-YF affects the activation of WT-STAT3 at multiple levels. Unexpectedly, the N-terminal domain of STAT3-YF plays an important role for the dominant-negative effect. We show that (i) STAT3-YF competes with WT-STAT3 in binding to activated gp130-receptors, (ii) the formation of WT-STAT3/STAT3-YF heterodimers in IL-6-stimulated cells results in inactive, semiphosphorylated dimers which do not bind to DNA and thus fail to induce target gene expression, (iii) the N-terminal domain-mediated formation of preformed STAT3/STAT3-YF heterodimers in unstimulated cells which affects the IL-6-induced homodimerization of WT-STAT3 contributes to the dominant-negative effect of STAT3-YF. These findings will contribute to our understanding of naturally occuring dominant-negative STAT3 mutants that cause the hyper-IgE syndrome.

## Background

The transcription factor STAT3 belongs to the signal transducer and activator of transcription (STAT) family which in mammals consists of seven proteins (STAT1, 2, 3, 4, 5A, 5B and 6). All STAT proteins are built up similarly. They consist of six conserved domains: a N-terminal domain followed by a coiled-coil domain, a DNA-binding domain, a linker domain, a SH2 domain and finally a transactivation domain. The phosphorylation of a specific tyrosine residue near the C-terminus is essential for STAT activation in response to cytokine stimulation [1,2].

In classical JAK/STAT signaling STAT proteins transmit the signal from receptors on the cell surface to the nucleus. IL-6-type cytokines which signal through the cytokine receptor subunit gp130 are among the most potent physiological STAT3 activators [3]. Ligand-binding to gp130 leads to activation of the associated Janus kinases (JAKs) which phosphorylate cytoplasmic tyrosine residues of the receptor. These phosphotyrosine residues serve as docking sites for molecules harboring a SH2 domain, such as STAT proteins. STAT3 is recruited to the receptor and becomes phosphorylated at tyrosine 705 by the activated JAKs. Phosphorylated STAT3 dimerizes by reciprocal phosphotyrosine/SH2 domain-interactions and translocates into the nucleus to induce target gene expression [4].

Beside the ligand-induced, phosphotyrosine-mediated dimerization of activated STAT3 there also exist so called preformed dimers in the absence of a stimulus [5,6]. Here, the dimerization is independent of phosphotyrosine/SH2 domain-interactions [7]. Similar to STAT1 the N-terminal domain of unphosphorylated STAT3 is essential for the formation of preformed STAT3 dimers in the absence of cytokine stimulation [8].

Among the seven mammalian STAT proteins STAT3 has the most pleiotropic functions [9]. Activation of STAT3 is sufficient to maintain the pluripotency of murine embryonic stem cells in vitro [10]. The early embryonic lethality observed in STAT3 knock-out mice illustrates a major role in embryonic development [11]. STAT3 is also an important transcription factor in immunity and inflammation. Therefore, STAT3 activation has to be tightly controlled. Dysregulated STAT3 signaling is linked to chronic inflammation and cancer and to the connection between these two diseases [12]. The protein SOCS3 (suppressor of cytokine signaling) is a well known target gene of STAT3 and acts as a classical feedback inhibitor [13].

Phosphorylation at tyrosine 705 is essential for STAT3 activation. Based on this fact, STAT3 Y705 point mutants should be inactive. STAT3-YF is an artificially generated STAT3 point mutant in which tyrosine 705 is substituted by phenylalanine [14]. The STAT3-YF mutant is often used for the analysis of cellular STAT3 functions that are independent of tyrosine 705 phosphorylation. Interestingly, STAT3-YF shows a pronounced dominant-negative effect on the activation of WT-STAT3 in stimulated cells through a molecular mechanism that is not completely understood. Other known dominant-negative forms of STAT3 are the splice-variant STAT3β lacking the transactivation domain, and several STAT3 point mutants which are linked to the autosomal-dominant hyper-IgE syndrome (HIES). In HIES, the amino acid exchanges are located in the DNA-binding or SH2-domain of STAT3. The dominant-negative activity of the mutants leads to an impaired function of STAT3 that results in a complex phenotype involving eosinophilia, eczema, recurrent skin and pulmonary infections and highly elevated IgE levels [15,16]. In this study we investigate the mechanism of STAT3-YF dominant-negative activity including gp130 receptor-binding as well as dimerization in the presence or absence of IL-6. We studied the relevance of the N-terminal domain for the dominant-negative activity of STAT3-YF. Understanding the dominant-negative activity of STAT3 mutants is potentially useful for the treatment of diseases like HIES.

## Results

### Dominant-negative STAT3-YF-YFP affects IL-6-induced SOCS3 gene expression and nuclear translocation of STAT3

**Figure 1:**
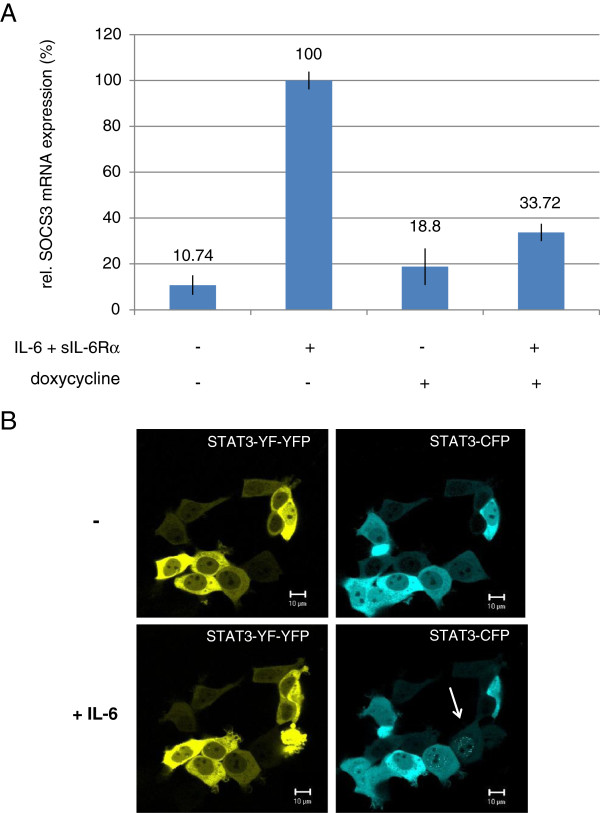
**Dominant-negative activity of STAT3-YF-YFP analyzed by real-time PCR and confocal microscopy. (A)** Real-time PCR analysis to examine the influence of STAT3-YF-YFP on the STAT3-induced SOCS3 mRNA expression after IL-6 stimulation. Inducible HEK cells stably transfected with STAT3-YF-YFP were incubated with 20 ng/ml doxycycline for 5 h and stimulated with 20 ng/ml IL-6 and 1 μg/ml sIL-6Rα for 60 minutes or left unstimulated. Non-induced HEK cells (without doxycycline) were treated the same way. RNA was isolated and SOCS3 mRNA expression analyzed by qRT-PCR. The diagram represents the mean of three independent measurements with standard deviations. **(B)** Nuclear translocation of STAT3-CFP in the presence of STAT3-YF-YFP in living cells after IL-6 stimulation. HEKgp80 cells were cotransfected with STAT3-YF-YFP and STAT3-CFP. Living cells were analyzed at 37°C by confocal microscopy. Pictures were taken from unstimulated cells and after stimulation with 20 ng/ml IL-6 for 60 minutes. The arrow marks the nuclear translocation of STAT3-CFP in a stimulated cell in the absence of STAT3-YF-YFP. Scale bars represent 10 μm.

For analysis of the dominant-negative STAT3-YF mutant we generated HEK cells which were stably transfected for inducible expression of a fluorescent STAT3-YF-YFP fusion protein (HEK-STAT3-YF-YFP cells). To demonstrate the dominant-negative activity we analyzed the effect of the STAT3-YF mutant on the endogenous wild-type STAT3 (WT-STAT3)-induced expression of SOCS3, a well known target gene of STAT3 (Figure 1A). Expression of STAT3-YF-YFP was induced with doxycycline and cells were stimulated with IL-6 and sIL-6Rα for 1 h. As a control HEK-STAT3-YF-YFP cells without doxycycline treatment were used. The IL-6-induced SOCS3 mRNA expression is strongly reduced in cells expressing STAT3-YF-YFP in comparison to cells without the mutant.

To identify the mechanism that causes reduced SOCS3 mRNA expression in the presence of STAT3-YF-YFP we analyzed the nuclear translocation of STAT3-YF and WT-STAT3 in living cells by confocal microscopy (Figure 1B). HEK cells stably expressing IL-6Rα (gp80) on the surface were transfected with STAT3-YF-YFP and STAT3-CFP. In non-stimulated cells STAT3-YF-YFP and STAT3-CFP are located predominantly in the cytoplasm. After stimulation with IL-6 STAT3-CFP translocated into the nucleus only in the absence of STAT3-YF-YFP expression (arrow in Figure 1B). The higher STAT3-YF-YFP is expressed, the lower is the STAT3-CFP nuclear translocation after IL-6 stimulation. Strong overexpression of STAT3-YF-YFP prevents nuclear accumulation of STAT3-CFP completely. Thus, the STAT3-YF mutant affects the nuclear translocation of WT-STAT3 in stimulated cells in a concentration-dependent manner. There is no nuclear accumulation of the STAT3-YF-YFP mutant after IL-6 stimulation detectable.

### STAT3-YF-YFP interferes with phosphorylation of endogenous STAT3 and binds to gp130 phosphopeptides

**Figure 2:**
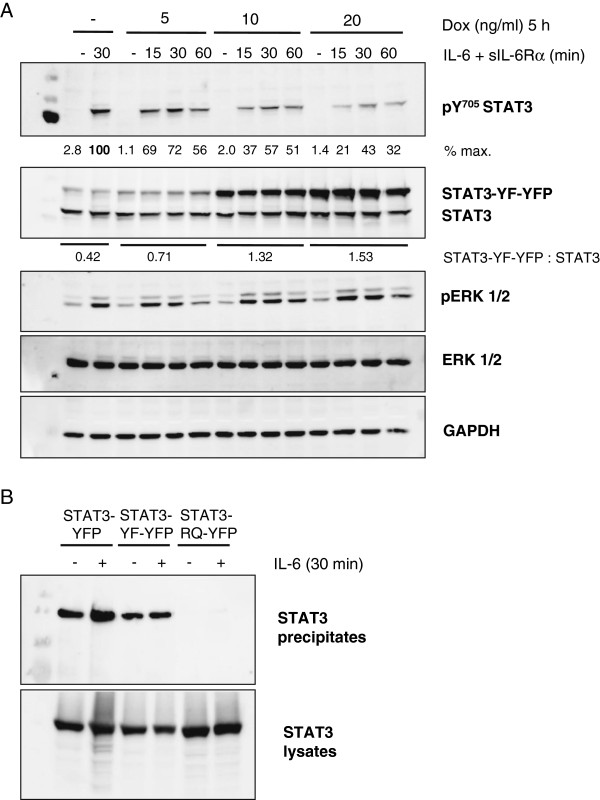
**Influence of STAT3-YF-YFP on the phosphorylation of endogenous STAT3 and analysis of binding to a gp130 phosphopeptide. (A)** Inducible HEK cells stably transfected with STAT3-YF-YFP were incubated with increasing concentrations of doxycycline (Dox) for 5 h. The cells were stimulated with 20 ng/ml IL-6 and 1 μg/ml sIL-6Rα for the indicated times or left unstimulated. Cellular lysates were analyzed by immunoblotting using antibodies against pY705-STAT3, STAT3, pERK1/2, ERK1/2 and GAPDH as a loading control. Signal intensities of STAT3, STAT3-YF-YFP and pY705 STAT3 were quantified. **(B)** HEKgp80 cells were transfected with STAT3-YFP, STAT3-YF-YFP or STAT3-R609Q-YFP. The cells were stimulated with 20 ng/ml IL-6 for 30 minutes or left unstimulated. The lysates were incubated with a biotinylated gp130-receptor-peptide containing phosphotyrosine 767 coupled to avidin-Sepharose. The precipitates were analyzed by immunoblotting using a specific antibody against STAT3. The expression of the transfected STAT3 constructs was verified by immunoblotting of untreated lysates.

STAT3 phosphorylation on tyrosine 705 is required for IL-6-induced nuclear accumulation of STAT3 and the induction of STAT3 target gene expression. Therefore, we examined the phosphorylation of endogenous WT-STAT3 in cells expressing STAT3-YF-YFP after stimulation with IL-6. HEK-STAT3-YF-YFP cells were incubated with increasing concentrations of doxycycline and stimulated with IL-6 and sIL-6Rα for different times. The cells were lysed and analyzed by immunoblotting (Figure 2A). Phosphorylation of endogenous STAT3 on tyrosine 705 is impaired by increasing levels of STAT3-YF-YFP in IL-6-stimulated cells in comparison to cells without the mutant. A 1.5-fold overexpression of STAT3-YF-YFP is sufficient to reduce tyrosine phosphorylation of endogenous STAT3 by more than 50% after 30 min of stimulation. As expected the STAT3-YF point mutant is not tyrosine phosphorylated after IL-6 stimulation. IL-6-induced ERK1/2 phosphorylation is not affected by STAT3-YF-YFP.

The reduced STAT3 phosphorylation in the presence of STAT3-YF-YFP might be caused by the binding of the STAT3-YF mutant to phosphorylated tyrosine residues of the activated gp130-receptor in IL-6-stimulated cells. We analyzed receptor-binding of the STAT3-YF point mutant by peptide-precipitation assays using a gp130-receptor-peptide containing the STAT3-recruiting phosphotyrosine residue 767. HEKgp80 cells were transfected with different STAT3 constructs and stimulated with IL-6 or left unstimulated. The peptide-precipitation samples were analyzed by immunoblotting (Figure 2B). The fluorescently labelled point mutant STAT3-YF-YFP binds to the gp130-receptor-peptide as WT-STAT3-YFP does. The binding is independent of IL-6 stimulation because of the phosphorylated peptide used. STAT3-R609Q-YFP harboring a mutation within the SH2 domain acts as a negative control. Binding of STAT3-YF-YFP to activated gp130-receptors could therefore compete with binding of WT-STAT3, leading to a reduced phosphorylation of WT-STAT3 as well as a reduced nuclear translocation and target gene expression.

### STAT3-YF-YFP forms heterodimers with WT-STAT3

**Figure 3:**
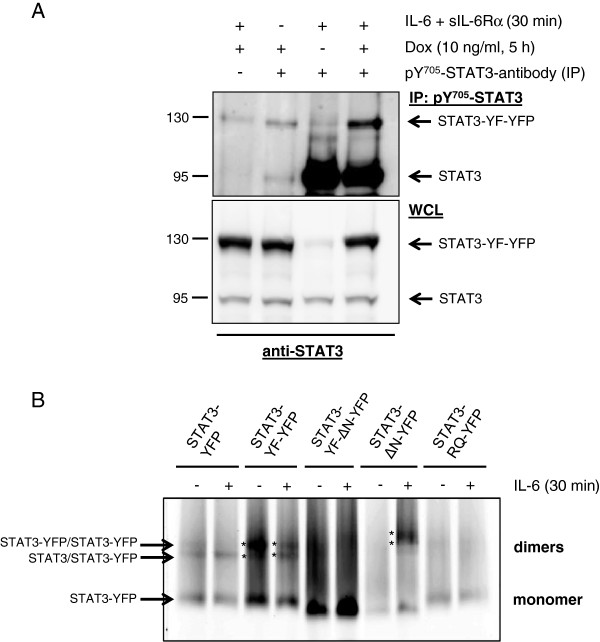
**Dimer-formation of STAT3-YF-YFP. (A)** Inducible HEK cells stably transfected with STAT3-YF-YFP were incubated with 10 ng/ml doxycycline (Dox) for 5 h or left untreated. The cells were stimulated with 20 ng/ml IL-6 and 1 μg/ml sIL-6Rα for 30 minutes or left unstimulated. The lysates were incubated with a specific antibody against an epitope comprising phosphotyrosine 705 of STAT3 coupled to Protein-G-Sepharose as indicated. Precipitates were analyzed by immunoblotting using a STAT3 antibody. Expression of the STAT3 constructs in the whole cell lysates (WCL) was verified by immunoblotting. **(B)** HEKgp80 cells were transfected with different YFP-labelled STAT3 constructs. The cells were stimulated with 20 ng/ml IL-6 for 30 minutes or left unstimulated. The lysates were incubated with Coomassie brilliant blue G-250 and separated on a 4-16% gradient gel under non-denaturing conditions. The gel was analyzed on a fluorescence scanner. Homo- as well as heterodimers are marked with arrows. Asterisks highlight STAT3-YF-YFP homodimers and WT-STAT3/STAT3-YF-YFP heterodimers as well as the corresponding homo- and heterodimers containing STAT3-ΔN-YFP.

In the classical JAK/STAT signaling pathway tyrosine phosphorylated STAT3 dimerizes after stimulation and translocates into the nucleus to induce target gene expression. We analyzed the heterodimerization of STAT3-YF-YFP with WT-STAT3 in IL-6-stimulated cells by immunoprecipitation. HEK-STAT3-YF-YFP cells were incubated with doxycycline and stimulated with IL-6 and sIL-6Rα. Since STAT3-YF-YFP is not tyrosine phosphorylated after stimulation we used an antibody against the epitope comprising phosphotyrosine 705 for specific precipitation of activated endogenous WT-STAT3. Precipitates were analyzed by immunoblotting (Figure 3A). Coprecipitation of STAT3-YF-YFP demonstrates an interaction of phosphorylated WT-STAT3 with STAT3-YF-YFP in IL-6-stimulated cells that does not require the accessibility of the phosphotyrosine motif (which is blocked by the precipitating antibody).

Based on this result we further tested the dimer-formation by blue-native polyacrylamide gel electrophoresis (BN-PAGE) under non-denaturing conditions. HEKgp80 cells were transfected with different YFP-labelled STAT3 constructs. Lysates of IL-6-stimulated and non-stimulated cells were separated by BN-PAGE and only the fluorescent proteins were detected by fluorescence-scanning of the gel (Figure 3B). In stimulated cells STAT3-YF-YFP homo- as well as heterodimers with WT-STAT3 were detectable (marked with asterisks). The same is visible in non-stimulated cells. These tyrosine phosphorylation-independent dimers are called preformed dimers and have been described previously [5,7]. As has been also described earlier, STAT3-ΔN only forms dimers upon phosphorylation [8] and STAT3-R609Q-YFP does not form dimers at all [17]. The interaction of STAT3-YF-YFP with WT-STAT3 might also be relevant for the dominant-negative effect. In this context, semiphosphorylated heterodimers after IL-6 stimulation as well as N-terminal domain mediated preformed dimers might be of importance.

### The N-terminal domain is required for the dominant-negative activity of STAT3-YF

To understand the importance of dimer-formation for the dominant-negative activity of STAT3-YF we focused on the N-terminal domain of STAT3-YF, because previous studies demonstrated that the N-terminal domain is essential for the formation of STAT3 dimers in the absence of a stimulus [8]. We generated a STAT3-YF-YFP construct lacking the N-terminal domain (STAT3-YF-ΔN-YFP) and tested the dimerization of the deletion mutant by BN-PAGE (Figure 3B). Independent of IL-6 stimulation only monomers were detected. When we transfected HEKgp80 cells with STAT3-ΔN-YFP, dimers were only detected in stimulated cells. This result confirms the importance of the N-terminal domain for the formation of preformed STAT3 dimers. Because of the point mutation at position 705 there are no STAT3-YF-ΔN-YFP dimers after IL-6 stimulation detectable in contrast to STAT3-ΔN-YFP which dimerizes after stimulation via phosphotyrosine/SH2 domain-interactions. As expected, the monomeric STAT3 deletion mutants migrate faster in the native gel than wild type STAT3-YFP or STAT3-YF-YFP. Compared to wild type STAT3-YFP or STAT3-YF-YFP the STAT3-ΔN-YFP dimer bands are shifted to apparent higher molecular masses (asterisks in Figure 3B). We attribute this irregular electrophoretic mobility to a less compact pSTAT3 dimer as the stabilization through the N-terminal domain is missing.

**Figure 4:**
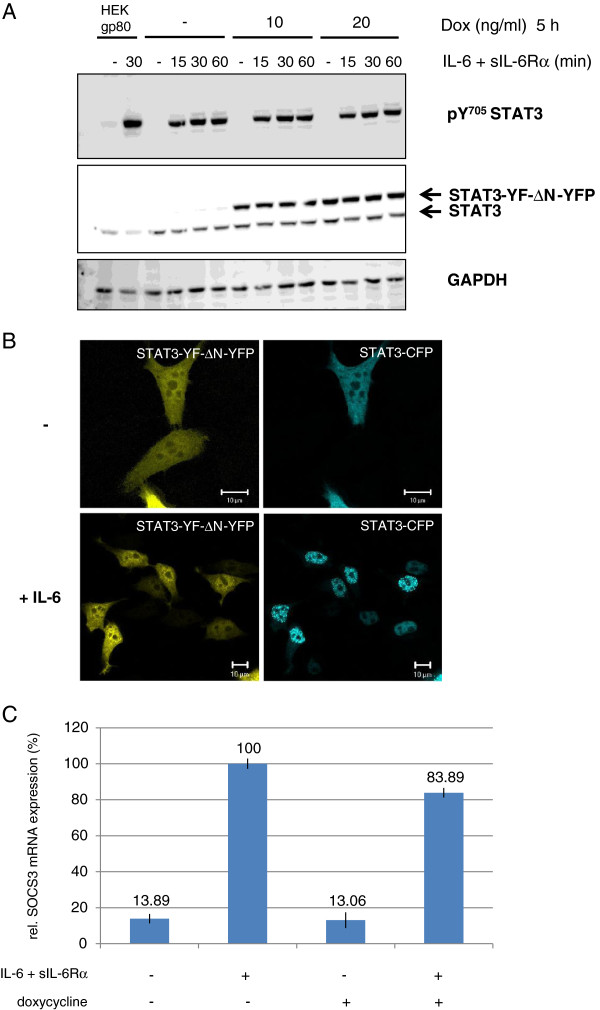
**Importance of the N-terminal domain for dominant-negative activity of the STAT3-YF mutant. (A)** Inducible HEK cells stably transfected with STAT3-YF-ΔN-YFP were incubated with 10 or 20 ng/ml doxycycline (Dox) for 5 h or left untreated. The cells were stimulated with 20 ng/ml IL-6 and 1 μg/ml sIL-6Rα for the indicated times or left unstimulated. HEKgp80 cells were stimulated with IL-6 for 30 minutes or left unstimulated. Cellular lysates were analyzed by immunoblotting using antibodies against pY705-STAT3, STAT3 and GAPDH as a loading control. **(B)** Nuclear translocation of STAT3-CFP in the presence of STAT3-YF-ΔN-YFP after IL-6 stimulation. HEKgp80 cells were cotransfected with STAT3-YF-ΔN-YFP and STAT3-CFP. The cells were stimulated with 20 ng/ml IL-6 for 30 minutes or left unstimulated. All cells were fixed and nuclear translocation was analyzed by confocal microscopy. Scale bars represent 10 μm. **(C)** Real-time PCR analysis to examine the influence of STAT3-YF-ΔN-YFP on the STAT3-induced SOCS3 mRNA expression after IL-6 stimulation. Inducible HEK cells stably transfected with STAT3-YF-ΔN-YFP were incubated with 20 ng/ml doxycycline for 5 h and stimulated with 20 ng/ml IL-6 and 1 μg/ml sIL-6Rα for 60 minutes or left unstimulated. Stably transfected HEK cells without doxycycline treatment served as a control. RNA was isolated and SOCS3 mRNA expression analyzed by qRT-PCR. The diagram represents the mean of three independent measurements with standard deviations.

Next we examined the dominant-negative activity of STAT3-YF-ΔN -YFP after IL-6 stimulation. HEK cells stably transfected with STAT3-YF-ΔN-YFP were incubated with increasing concentrations of doxycycline and stimulated with IL-6 and sIL-6Rα for different times. The cell lysates were analyzed by immunoblotting (Figure 4A). In IL-6-stimulated cells expressing STAT3-YF-ΔN-YFP no reduced tyrosine 705 phosphorylation of endogenous STAT3 is detectable (compare with Figure 2A). The IL-6-induced nuclear translocation of STAT3-CFP in the presence of STAT3-YF-ΔN-YFP was analyzed by confocal microscopy in fixed cells (Figure 4B). In non-stimulated HEKgp80 cells STAT3-YF-ΔN-YFP and STAT3-CFP are located in the cytoplasm as well as in the nucleus. After IL-6 stimulation STAT3-CFP translocates into the nucleus independently of the expression level of STAT3-YF-ΔN-YFP. To investigate the dominant-negative activity on gene induction we tested the influence of STAT3-YF-ΔN-YFP on WT-STAT3-induced SOCS3 expression by real-time PCR (Figure 4C). HEK cells stably transfected with STAT3-YF-ΔN-YFP were incubated with doxycycline and stimulated with IL-6 and sIL-6Rα for 1 h. STAT3-YF-ΔN-YFP only marginally interferes with SOCS3 mRNA expression in comparison to non-induced cells that do not express STAT3-YF-ΔN-YFP.

The results demonstrate that the N-terminal domain is essential for the dominant-negative effect of STAT3-YF. STAT3-YF-YFP lacking the N-terminal domain forms no dimers, neither with unphosphorylated nor with phosphorylated WT-STAT3. The mutant does not interfere with phosphorylation and nuclear translocation of WT-STAT3 and therefore hardly affects the induction of SOCS3 mRNA expression.

### Preformed dimers of STAT3-YF-YFP-NLS interfere with STAT3-DNA-binding

**Figure 5:**
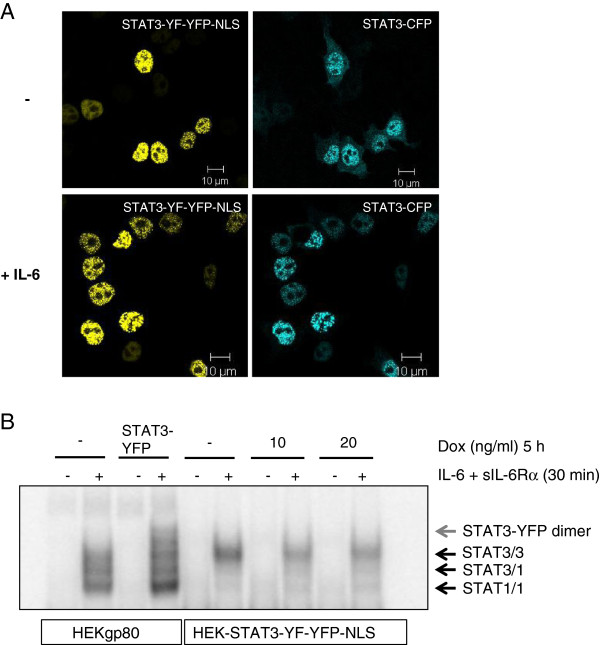
**The nuclear function of the STAT3-YF mutant*. *(A)** HEKgp80 cells were cotransfected with STAT3-YF-YFP-NLS and STAT3-CFP. The cells were stimulated with 20 ng/ml IL-6 for 30 minutes or left unstimulated. All cells were fixed and nuclear translocation was analyzed by confocal microscopy. Scale bars represent 10 μm. **(B)** HEK cells stably transfected with STAT3-YF-YFP-NLS were incubated with 10 or 20 ng/ml doxycycline (Dox) for 5 h or left untreated. The cells were stimulated with 20 ng/ml IL-6 and 1 μg/ml sIL-6Rα for 30 minutes or left unstimulated. Non-transfected or STAT3-YFP transfected HEKgp80 cells were stimulated the same way. Nuclear extracts were prepared and analyzed in an electrophoretic mobility shift assay using hybridized ^32^P-labelled oligonucleotides that form a STAT3 binding site.

To study how dominant-negative activity of STAT3-YF-YFP depends on the subcellular localization, we generated a STAT3-YF-YFP construct with a NLS sequence (STAT3-YF-YFP-NLS). The distribution of the mutant together with WT-STAT3 was analyzed by confocal microscopy in fixed HEKgp80 cells (Figure 5A). In non-stimulated cells STAT3-YF-YFP-NLS is clearly located in the nucleus. Interestingly, WT-STAT3-CFP accumulates in the nucleus together with STAT3-YF-YFP-NLS. This finding strongly supports the notion of preformed dimers between STAT3-YF and WT-STAT3 in the absence of IL-6 stimulation. After IL-6 stimulation STAT3-CFP completely translocates into the nucleus because nuclear STAT3-YF-YFP-NLS cannot interfere with receptor-mediated phosphorylation of WT-STAT3-CFP. The dominant-negative activity of STAT3-YF-YFP-NLS on SOCS3 gene induction was verified by real-time PCR (data not shown).

Because of the nuclear dominant-negative activity we examined DNA-binding of the STAT3-YF-YFP-NLS mutant in stably transfected, inducible HEK cells by electrophoretic mobility shift assay (EMSA, Figure 5B). After IL-6 stimulation there are no DNA-bound STAT3-YF-YFP-NLS dimers detectable, but STAT3-homodimers. DNA-binding of WT-STAT3 is reduced in cells expressing STAT3-YF-YFP-NLS in comparison to non-induced cells. Although STAT3-YF forms preformed homodimers as well as heterodimers with WT-STAT3 there is no DNA-binding visible in stimulated cells. Instead the presence of the mutant interferes with the binding of WT-STAT3 to DNA most probably as a result of heterodimer formation between WT-STAT3 and STAT3-YF mutant.

## Discussion

The STAT3-YF point mutant acts in a dominant-negative manner on WT-STAT3 activity in stimulated cells. Here we studied the molecular mechanism of STAT3-YF activity in IL-6-stimulated cells and analyzed the importance of the N-terminal domain for the dominant-negative effect.

Nakajima et al. previously described that STAT3 activity in IL-6-stimulated cells is impaired in the presence of STAT3-YF [18]. The reduced WT-STAT3-induced SOCS3 mRNA expression in IL-6-stimulated HEK-STAT3-YF-YFP cells shown here confirms this finding. STAT3-YF-YFP affects phosphorylation, nuclear translocation and transcriptional activity of endogenous WT-STAT3. In the classical JAK/STAT pathway the binding of STAT3 to activated receptors followed by Y705 phosphorylation is the first step to induce STAT3 signal transduction. STAT3-YF-YFP binds to a tyrosine-phosphorylated gp130-receptor-peptide comparable to WT-STAT3-YFP. Kaptein et al. previously described that STAT3-YF may compete with STAT3 for binding to phosphotyrosine residues of gp130-receptors and when in excess exclude STAT3 recruitment [14]. Our findings support this assumption. Thus, we conclude that a competition in binding to STAT3-recruiting phosphotyrosine residues of activated gp130-receptors in IL-6-stimulated cells contributes to the dominant-negative activity of STAT3-YF. As expected the STAT3-YF mutant is not tyrosine phosphorylated and does not accumulate in the nucleus of IL-6-stimulated cells.

Another possible mechanism of STAT3-YF activity proposed by Kaptein and coworkers is based on the formation of non-functional weak heterodimers with activated STAT3 [14]. Immunoprecipitations using an antibody against the phosphotyrosine 705 peptide motif of STAT3 and BN-PAGE shown in this study strengthen the hypothesis of semiphosphorylated WT-STAT3/STAT3-YF heterodimers. The WT-STAT3/STAT3-YF heterodimers are inactive and not able to bind to DNA after IL-6 stimulation as analyzed here with HEK-STAT3-YF-YFP-NLS cells. Instead the presence of the mutant impairs the DNA-binding of active WT-STAT3 probably as a result of heterodimer formation between WT-STAT3 and STAT3-YF. The STAT3-YF mutant forms dimers in the presence and absence of IL-6 comparable to WT-STAT3. The preformed, unphosphorylated dimers were also detectable in unstimulated cells cotransfected with STAT3-YF-YFP-NLS and WT-STAT3-CFP. Kretzschmar et al. also detected STAT3/STAT3-YF heterodimers by FRET-analysis in unstimulated and stimulated cells [17]. The heterodimers seem to be inactive because after stimulation no phosphotyrosine/SH2 domain-interactions were detectable, which is essential for the formation of active, DNA-binding dimers. Based on these results, we conclude that the formation of inactive, semiphosphorylated STAT3/STAT3-YF dimers is another molecular mechanism for the dominant-negative activity of STAT3-YF.

**Figure 6:**
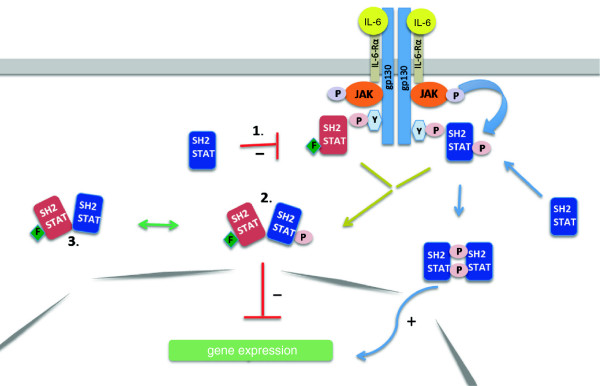
**The dominant-negative effect of STAT3-YF in the IL-6-induced STAT3 signaling pathway.** The dominant-negative effect of STAT3-YF (red) on the WT-STAT3 (blue) activity in the cells relies on: 1) Competition in binding to phosphotyrosine motifs of the activated gp130-receptor 2) Formation of inactive, semiphosphorylated, non-DNA-binding heterodimers after IL-6 stimulation 3) Formation of preformed dimers between STAT3-YF and WT-STAT3 in the absence of IL-6 which reduces the capability of STAT3 to form active, DNA-binding homodimers after stimulation.

Our data suggest that preformed heterodimers of STAT3-YF and WT-STAT3 also play an essential role for the dominant-negative effect. We previously described the importance of the N-terminal domain for the formation of unphosphorylated preformed STAT3 dimers in non-stimulated cells [8]. As shown here STAT3-YF-ΔN-YFP forms dimers neither in unstimulated nor in IL-6-stimulated cells. Interestingly, only a weak dominant-negative effect of STAT3-YF-ΔN-YFP is detectable in IL-6-stimulated cells in comparison to full-length STAT3-YF-YFP. The group of Vinkemeier previously described a possible protein-interaction via the N-terminal domains in semiphosphorylated STAT1 heterodimers because a single Y701 phosphorylation might result only in weak and instable STAT1 dimers [19]. The same interaction through the N-terminal domains is also conceivable for semiphosphorylated STAT3/STAT3-YF heterodimers. Thus, there should be no prominent conformational changes between preformed STAT3/STAT3-YF dimers and semiphosphorylated dimers after stimulation. Our assumption is in line with results of FRET-measurements by Kretzschmar et al. [17] and is contrary to Kapteins model [14] which describes a weak heterodimerization via a single phosphotyrosine/SH2 domain-interaction. Additionally, preformed STAT3/STAT3-YF heterodimers reduce the chance of WT-STAT3 to find another STAT3 protein which is phosphorylated on Y705. In this way preformed STAT3/STAT3-YF heterodimers prevent the formation of active STAT3 homodimers. We thus conclude that N-terminal domain-dependent heterodimer formation with WT-STAT3 significantly contributes to the dominant-negative activity of the STAT3-YF mutant (Figure 6). The presented molecular mechanism of STAT3-YF activity might also be relevant for the understanding of several dominant-negative STAT3 point mutants which are linked to autosomal-dominant HIES. In a recent study it has been found that STAT3 mutated in the DNA-binding domain is still phosphorylated on Y705 in response to IL-6. However, SH2-domain mutants are not phosphorylated anymore [20]. For these mutants an interaction through the N-terminal domains might also be required for dominant-negative activity. In these cases, blockade of N-terminal domain interactions could be an option for future treatment of the disease.

## Conclusions

The strong dominant-negative activity of the STAT3-Y705F mutant results from its interference with WT-STAT3 activity at multiple levels including (i) activation at cytokine receptors (ii) formation of inactive heterodimers with phosphorylated WT-STAT3 and (iii) formation of heterodimers with unphosphorylated WT-STAT3. The latter two activities require the N-terminal domain which is indispensable for the formation of unphosphorylated STAT3 dimers. These findings have some implications for the understanding and targeting of naturally occurring STAT3 mutants with dominant-negative activity that cause the hyper-IgE syndrome.

## Methods

### Cytokines and cytokine receptors

Recombinant human IL-6 was expressed in *Escherichia coli*, refolded and purified as described previously [21]. The soluble IL-6Rα (sIL-6Rα) was expressed in insect cells as previously described [22].

### Cell culture and transfection

Human embryonic kidney cells stably expressing IL-6Rα on the cell surface (HEKgp80) (kind gift of Dr. Anna Dittrich, Otto von Guericke-University, Magdeburg, Germany) and different inducible stably transfected STAT3-YF HEK cells were grown in Dulbecco’s Modified Eagle Medium (DMEM) with Glutamax™ supplemented with 10% fetal calf serum (FCS, PAA, Germany), 60 mg/l penicillin, 100 mg/l streptomycin and 2 mg/l puromycin (Gibco, Germany). The cells were incubated at 37°C in a water saturated atmosphere at 5% CO_2_. Flp-In T-REx™-293 cells were stably transfected with STAT3-YF-YFP, STAT3-YF-ΔN-YFP or STAT3-YF-YFP-NLS using the Flp-In™ system (Invitrogen, UK), ensuring integration of the construct and induction of gene expression with doxycycline. Transient transfections were performed by using TransIT-LT-1 transfection reagent (Mirus, USA) according to the manufacturer’s instruction.

### Preparation of cell lysates, SDS-PAGE and immunoblotting

Inducible stably transfected STAT3-YF-YFP or STAT3-YF-ΔN-YFP HEK cells were incubated with increasing concentrations of doxycycline for 5 h. The cells were stimulated with 20 ng/ml IL-6 and 1 μg/ml sIL-6Rα for different times or left unstimulated. Afterwards the cells were washed with PBS (150 mM NaCl, 2.5 mM KCl, 8.0 mM Na_2_HPO_4_, 1.5 mM KH_2_PO_4_, adjusted to pH 7.4) and lysed with RIPA lysis buffer (50 mM Tris–HCl pH 7.4, 150 mM NaCl, 1 mM EDTA, 0.5% Nonidet P40, 1 mM NaF, 15% glycerol) supplemented with phosphatase/protease inhibitors (1 mM Na_3_VO_4_, 0.25 mM phenylmethylsulfonylfluoride (PMSF), 0.5 mM EDTA, 5 μg/ml aprotinin and 1 μg/ml leupeptin). Protein concentrations of the supernatants were measured using the Bio-Rad protein assay (Bio-Rad, Germany). The lysates were analyzed by SDS-PAGE, Western blotting and immunodetection using specific antibodies directed against pY705-STAT3 (Cell signaling, USA), STAT3 (BD transduction laboratories and Santa Cruz Biotechnology, USA), pERK1/2 (Cell signaling, USA), ERK1/2 (Santa Cruz Biotechnology, USA), GAPDH (Santa Cruz Biotechnology, USA) and horseradish-peroxidase conjugated secondary antibodies (DAKO, Denmark). All primary antibodies were used in a 1:1000 dilution, all secondary antibodies in a 1:2000 dilution in TBS-N buffer (20 mM Tris–HCl pH 7.6, 140 mM NaCl and 0.1% Nonidet P40). Bound antibodies were detected by enhanced chemiluminescence (ECL; Millipore, USA).

### Preparation of nuclear extracts and electrophoretic mobility shift assay (EMSA)

Inducible stably transfected STAT3-YF-YFP-NLS HEK cells were incubated with 10 or 20 ng/ml doxycycline for 5 h or left untreated. The cells were stimulated with 20 ng/ml IL-6 and 1 μg/ml sIL-6Rα for 30 minutes or left unstimulated. Non-transfected or STAT3-YFP transfected HEKgp80 cells were stimulated with 20 ng/ml IL-6 for 30 minutes or left unstimulated. Afterwards all cells were washed twice and harvested with PBS containing sodium vanadate. The pellet was resuspended in buffer A (10 mM Hepes-KOH pH 7.9, 1.5 mM MgCl_2_, 10 mM KCl, 0.5 mM DTT, 0.2 mM PMSF, 1 mM sodium vanadate), incubated for 10 minutes on ice and centrifuged for 10 seconds at 16,000 *g*. The pellet was resuspended in buffer C (20 mM Hepes-KOH pH 7.9, 420 mM NaCl, 1.5 mM MgCl_2_, 0.2 mM EDTA, 25% glycerol, 0.5 mM DTT, 0.2 mM PMSF, 1 mM sodium vanadate), incubated 20 minutes on ice and centrifuged for 2 minutes at 16,000 *g*. Protein concentrations of the supernatants were measured using the Bio-Rad protein assay (Bio-Rad, Germany). A double-stranded mutated *sis*-inducible element (SIE) oligo-nuleotide from the *c-fos* promotor (m67SIE: 5′-GATCCGGGAGGGATTTACGGGAAATGCTG-3′) was labelled by filling in 5′- protruding ends with the Klenow enzyme using [α-^32^P]dATP. Nuclear extracts containing 5 μg protein were incubated with about 10 fmol (10,000 cpm) of labelled oligonucleotides in gel shift incubation buffer (10 mM Hepes-KOH pH 7.8, 1 mM EDTA, 5 mM MgCl_2_, 10% glycerol, 5 mM DTT, 2 mM PMSF, 0.05 mg/ml of poly(dI-dC) and 1 mg/ml BSA) for 10 minutes at room temperature. The DNA–protein complexes were separated on a 4.5% polyacrylamide gel containing 7.5% glycerol in 0.25-fold TBE buffer (200 mM Tris, 166 mM boric acid, 2 mM EDTA, adjusted to pH 8.3) at 20 V/cm. The gel was fixed in 10% methanol and 10% acetic acid for 15 minutes, dried and analyzed by autoradiography.

### Blue-native polyacrylamide gel electrophoresis (BN-PAGE)

HEKgp80 cells transiently transfected with different YFP-labelled STAT3 constructs were stimulated with 20 ng/ml IL-6 for 30 minutes or left unstimulated. The cells were lysed by using the NativePAGE™ Sample Prep Kit (Invitrogen, UK) with DDM (n-dodecyl-beta-maltoside) as detergent in a final concentration of 10 g/l. The lysates were mixed with Coomassie brilliant blue G-250 and separated on a 4-16% gradient gel using the NativePAGE™ Novex Bis-Tris gel system (Invitrogen, UK). The protein complexes were separated over night at 4°C. YFP fluorescence was detected with a typhoon gel fluorescence scanner (GE Healthcare, Germany) by excitation with a 488 nm laser line. The emission was detected using a 515–555 nm bandpass filter.

### Peptide-precipitation

HEKgp80 cells transiently transfected with STAT3-YFP, STAT3-YF-YFP or STAT3-R609Q-YFP were stimulated with 20 ng/ml IL-6 for 30 minutes or left unstimulated. The cells were lysed with storage buffer (150 mM NaCl, 50 mM Tris–HCl pH 7.5, 0.1 mM EDTA, 10% glycerin, 0.5% Nonidet P40) supplemented with phosphatase/protease inhibitors. 3 nmol of a biotinylated gp130-receptor-peptide containing the STAT3 recruiting phosphotyrosine residue 767 (biotin-betaA-TVVHSGY(767)RHQV-PSV) was incubated with 10 μl NeutrAvidin Agarose (Pierce, USA) in PBS for 1 h at 4°C. The Sepharose was washed twice with storage buffer and incubated with 250 μg cell lysate in a final volume of 500 μl over night at 4°C. After incubation the samples were washed three times with storage buffer for 10 minutes and centrifuged for 1 minute at 4°C. The precipitates were analyzed by SDS-PAGE, Western blotting and immunodetection.

### Immunoprecipitation

Protein-G-Sepharose (GE Healthcare, Germany) was incubated with a specific antibody directed against pY705-STAT3 (Cell signaling, USA) over night at 4°C. Inducible stably transfected STAT3-YF-YFP HEK cells were incubated with 10 ng/ml doxycycline for 5 h. Afterwards the cells were stimulated with 20 ng/ml IL-6 and 1 μg/ml sIL-6Rα for 30 minutes or left unstimulated. The cells were lysed with RIPA buffer (50 mM Tris–HCl pH 7.4, 150 mM NaCl, 1 mM EDTA, 0,5% NP-40 (IGEPAL), 1 mM NaF, 15% glycerol) supplemented with phosphatase/protease inhibitors. The Sepharose was washed three times with RIPA and incubated with the cell lysates over night at 4°C. The precipitates were washed three times with RIPA and analyzed by SDS-PAGE, Western blotting and immunodetection.

### Quantitative real-time PCR (qRT-PCR)

Inducible stably transfected STAT3-YF-YFP or STAT3-YF-ΔN-YFP HEK cells were incubated with 20 ng/ml doxycycline for 5 h or left untreated. The cells were stimulated with 20 ng/ml IL-6 and 1 μg/ml sIL-6Rα for 60 minutes or left unstimulated. Total RNA was isolated using the RNeasy Mini Kit (QIAGEN, Germany) according to the manufacturer’s instructions. RNA was reverse transcribed into complementary DNA (cDNA) using the Omniscript-RT-PCR Kit (QIAGEN, Germany). TaqMan gene expression assays for human SOCS3 (Hs00269575_s1) and human HPRT (Hs99999909_m1) as internal standard were obtained from Applied Biosystems (Carlsbad, USA), and polymerase chain reaction (PCR) was performed using qPCR Mastermix plus (Eurogentec, Germany). The PCR reaction was done in a final volume of 20 μl containing 2 μl cDNA and 1 μl TaqMan gene expression assay solution using a Rotorgene (QIAGEN, Germany). Amplification of cDNA was initiated with a 2 min incubation at 50°C and 10 min at 95°C followed by 40 cycles of 95°C for 15 s and 60°C for 60 s. The gene of interest and the HPRT gene were amplified in duplicates. The quantification of gene expression was calculated using the Pfaffl method [23].

### Confocal fluorescence microscopy

Confocal imaging was performed with a Zeiss LSM 510 Meta confocal microscope (Zeiss, Germany). CFP fluorescence was detected using a 458 nm line of the argon laser and a bandpass filter BP 480/20 nm. YFP fluorescence was detected using a 514 nm line of the argon laser and a 530–600 nm bandpass filter. The images shown represent confocal slices of approximately 1 μm. The cells were examined with a 63×/1.2 NA water immersion objective. For fixation, transiently transfected HEKgp80 cells were grown on glass coverslips. The cells were stimulated with 20 ng/ml IL-6 for 30 minutes or left unstimulated. Afterwards the cells were fixed with 3.7% paraformaldehyde for 20 minutes and washed twice with PBS containing 1 mM MgCl_2_ and 0.1 mM CaCl_2_ (PBS++). The cells were quenched with 50 mM NH_4_Cl in PBS++ for 5 minutes, dipped in water and mounted with ImmuMount (Thermo Scientific, UK).

For live-cell imaging, transiently transfected HEKgp80 cells were grown on 42-mm glass coverslips. The coverslips were placed into a thermostatted (37°C) and CO_2_-controlled incubation chamber (Pecon, Germany) with fresh medium (Invitrogen). Pictures were taken in the absence of a stimulus and after stimulation of the cells with 20 ng/ml IL-6 for different times.

## Competing interests

The authors declare that they have no competing interests.

## Authors’ contributions

AM has performed most of the depicted experiments, interpreted the data and wrote a first draft version of the manuscript. DF and NR have studied the interaction of STAT3 with STAT3-YF and contributed significantly to the final manuscript. GMN has initiated and designed the study, interpreted the data and critically revised the manuscript. All authors have read and approved the final manuscript.
